# TargetCOPD: a pragmatic randomised controlled trial of targeted case finding for COPD *versus* routine practice in primary care: protocol

**DOI:** 10.1186/1471-2466-14-157

**Published:** 2014-10-04

**Authors:** Rachel E Jordan, Peymané Adab, Sue Jowett, Jen L Marsh, Richard D Riley, Alexandra Enocson, Martin R Miller, Brendan G Cooper, Alice M Turner, Jon G Ayres, Kar Keung Cheng, Kate Jolly, Robert A Stockley, Sheila Greenfield, Stanley Siebert, Amanda Daley, David A Fitzmaurice

**Affiliations:** School of Health and Populations Sciences, University of Birmingham, Birmingham, UK; Lung Investigation Unit, University Hospitals Birmingham NHS Foundation Trust, Birmingham, UK; Queen Elizabeth Hospital Research Laboratories, Mindelsohn Way, Birmingham, UK; Business School, University of Birmingham, Birmingham, UK; Public Health, Epidemiology & Biostatistics, Public Health Building, School of Health and Population Sciences, University of Birmingham, Edgbaston, Birmingham B15 2TT UK

**Keywords:** COPD, Case-finding, Screening, Primary care, Respiratory questionnaire, Spirometry, Cluster RCT, Cost-effectiveness

## Abstract

**Background:**

Many people with clinically significant chronic obstructive pulmonary disease (COPD) remain undiagnosed worldwide. There are a number of small studies which have examined possible methods of case finding through primary care, but no large RCTs that have adequately assessed the most cost-effective approach.

**Methods/Design:**

In this study, using a cluster randomised controlled trial (RCT) in 56 general practices in the West Midlands, we plan to investigate the effectiveness and cost-effectiveness of a Targeted approach to case finding for COPD compared with routine practice. Using an individual patient RCT nested in the Targeted arm, we plan also to compare the effectiveness and cost-effectiveness of Active case finding using a postal questionnaire (with supplementary opportunistic questionnaires), and Opportunistic-only case finding during routine surgery consultations.

All ever-smoking patients aged 40-79 years, without a current diagnosis of COPD and registered with participating practices will be eligible. Patients in the Targeted arm who report positive respiratory symptoms (chronic cough or phlegm, wheeze or dyspnoea) using a brief questionnaire will be invited for further spirometric assessment to ascertain whether they have COPD or not. Post-bronchodilator spirometry will be conducted to ATS standards using an Easy One spirometer by trained research assistants.

The primary outcomes will be new cases of COPD and cost per new case identified, comparing targeted case finding with routine care, and two types of targeted case finding (active *versus* opportunistic). A multilevel logistic regression model will be used to model the probability of detecting a new case of COPD for each treatment arm, with clustering of patients (by practice and household) accounted for using a multi-level structure.

A trial-based analysis will be undertaken using costs and outcomes collected during the trial. Secondary outcomes include the feasibility, efficiency, long-term cost-effectiveness, patient and primary care staff views of each approach.

**Discussion:**

This will be the largest RCT of its kind, and should inform how best to identify undiagnosed patients with COPD in the UK and other similar healthcare systems. Sensitivity analyses will help local policy-makers decide which sub-groups of the population to target first.

**Trial registration:**

Current controlled trials ISRCTN14930255

**Electronic supplementary material:**

The online version of this article (doi:10.1186/1471-2466-14-157) contains supplementary material, which is available to authorized users.

## Background

Chronic obstructive pulmonary disease (COPD) affects 5-10% of adults worldwide [1], is rising in prevalence [2], and is a leading cause of death [3]. In the UK it accounts for 1.4 million GP consultations, 1 million in-patient days per year and costs the NHS over £800 million per year [4]. However, around 45-85% of cases [5–7], depending on diagnostic criteria, remain undiagnosed, representing many with potentially unmet need. There is much uncertainty about how to approach early identification of patients [8, 9], although it has been recently demonstrated that there are many missed opportunities to diagnose patients in primary care where signs and symptoms have been overlooked [10].

The CMO's report, 2005 [11] and the Healthcare Commission report [12] highlighted the burden of COPD, the extent of under-diagnosis and variation in access to relevant services. As a result a National Clinical Outcomes Strategy has been published recommending case finding in high risk patients [13], resulting in a British Lung Foundation campaign [14] and drive to identify missing cases [15, 16]. Several NHS Trusts have started initiatives [17, 18].

Despite the move towards early case-finding for COPD, the most effective and cost-effective approach is not known. There are no relevant published systematic reviews, though many small pre-post studies have reported on approaches to case finding. These report either active [5, 19, 20] or opportunistic [21–24] approaches (with yield 18-47%), but are limited because of a lack of comparison groups, restricted number and range of participants, incomplete follow up or different target populations. More recently a few trials have been published. A small trial from a single practice reported a higher prevalence of airflow obstruction with an opportunistic compared to invitation based approach to case finding [25]. The population however was highly deprived, with many non-English speakers, and no economic analysis was undertaken. A cluster RCT in 16 general practices in the Netherlands which used an initial screening questionnaire to identify patients at risk found that a practice-managed approach to calculating the score and inviting patients for spirometry assessment was more effective than a patient-led approach of applying for spirometry assessment having calculated their own scores [26]. Additionally, we have also reported a pilot study in two general practices which suggested that it may be more cost-effective to undertake opportunistic screening; however the study was underpowered and the results require proper testing in a full RCT [27]. The limitation of the current evidence base and lack of adequate data from RCTs contributed to the recent recommendation by the UK National Screening Committee against screening for COPD [28], and justifies a new adequately-powered trial.

Many studies have used spirometry alone to screen for COPD. However this is not recommended [8] as it would identify many without clinically important disease for whom there is little evidence of effective interventions [29, 30]. National Institute for Clinical Excellence (NICE) guidelines recommend opportunistic case finding in those presenting with relevant symptoms and exposures (mainly smoking) [31]. Our analysis of the Health Survey for England (HSE), a large dataset representative of the English population, shows that targeted case-finding among smokers aged ≥40 with relevant symptoms would have greater yield than targeting the general population [32]. Of the undiagnosed cases likely to be detected, more than three quarters could benefit from evidence based treatment (e.g. pulmonary rehab, inhaled therapies, vaccination & smoking cessation interventions), and there is new evidence that others may also benefit [33]. Modelling of data from the Health Survey for England indicated that active case-finding (with postal questionnaire plus opportunistic case-finding in primary care consultations) would be more effective than opportunistic case-finding alone, but this needs empirical testing [32].

Cases identified are expected to benefit from treatment resulting in improved quality of life, increased survival, reduction in hospital admissions and less work-related absence. Published economic evaluations in COPD have primarily considered interventions for the disease rather than diagnosis, and others have concentrated on the costs of COPD in burden of illness studies. No trial-based economic evaluation has considered case-finding. NICE guidelines included simple decision-tree based modelling to determine the cost-effectiveness of opportunistic case finding among ever smokers over 35 years old with chronic cough [31]. The model is simplistic, contains many assumptions and requires better data on the natural history of the disease. The proposed cost-effectiveness component of this study will resolve this issue.

In this study, using a cluster RCT in general practices across the West Midlands, we plan to investigate the effectiveness and cost-effectiveness of a Targeted approach to case finding for COPD compared with routine practice. Using an individual patient RCT nested in the targeted arm, we plan also to compare the effectiveness and cost-effectiveness of Active case finding using a postal questionnaire (with supplementary opportunistic questionnaires), and Opportunistic case finding at routine surgery consultations.

## Methods

### Aims and objectives

- To determine whether Targeted case finding is more effective and cost-effective than routine practice.
- To evaluate the relative effectiveness and cost-effectiveness of two alternative methods of targeted case finding: Active (postal questionnaire plus flagging of patient records) versus Opportunistic (flagging only).

### Design

**Figure 1:**
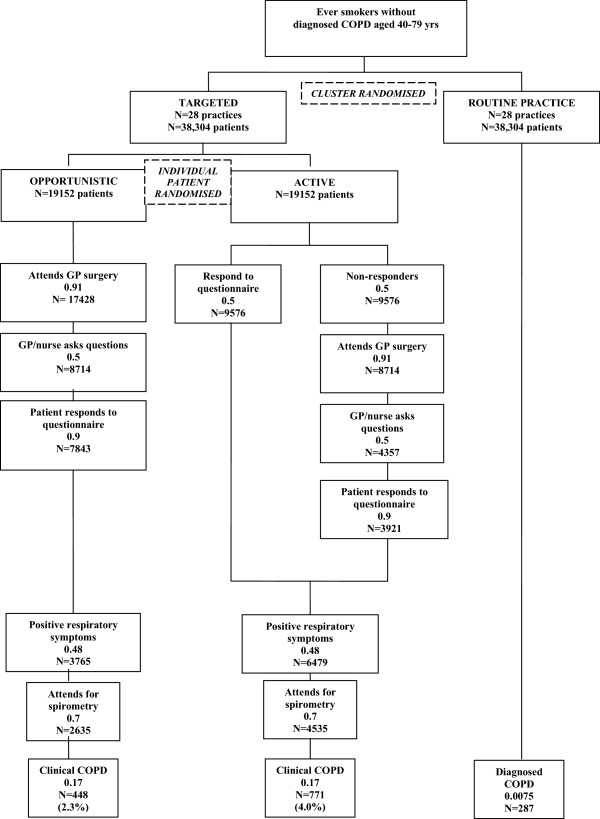
**Plan of trial including projected numbers** [32] **.**

TargetCOPD is a pragmatic randomised controlled trial of a case-finding intervention, delivered at organisational level, with two elements (Figure 1):

1. Cluster randomised trial comparing Targeted case finding (28 practices) with routine practice (28 practices).
2. Individually randomized controlled trial nested within the targeted case finding arm comparing two alternative types of targeting: Active (A) versus Opportunistic (O).

### Setting

Fifty six general practices in the West Midlands will be recruited through the Primary Care Research Network for Central England (PCRN-CE) at the University of Birmingham.

### Participants

Patients will be identified as eligible if they are:

- registered at a participating practice
- aged between 40 and 79 years (inclusive) on the date of the record search,
- identified as current or ex-smokers according to their patient records
- have no prior diagnosis of COPD

Automated computer searches will be used for identifying all eligible patients using READ codes (Additional file 1: Appendix 1). Patients will be further assessed for eligibility to take part in assessments by their GP and those not considered suitable will not be invited for assessment (e.g. if unable to give informed consent, suffering from a terminal illness or pregnant).

### Intervention

All interventions will take place over one year. The schema is given in Figure 1 with projected patient numbers. Fifty six practices will initially be randomised to continue with routine care or to a targeted approach to case finding.

#### Routine practice arm

In the routine practice arm of the cluster trial, practices will continue with their usual care and new cases of COPD will be identified according to usual practice. NICE guidance recommends that patients over the age of 35 years should be investigated for COPD with spirometry if they present opportunistically with chronic cough or phlegm [31].

#### Targeted case finding arm

Within the targeted arm of the cluster trial, participants will be randomly allocated (by household) to receive either the Opportunistic or the Active case finding intervention:

#### Stage 1: respiratory questionnaire

##### Opportunistic

Patients in this arm will have their records flagged to prompt the consulting healthcare practitioner or receptionist to provide them with a brief screening questionnaire consisting of simple respiratory questions supplemented with basic socio-demographic and medical information (Additional file 1: Appendix 2). The questionnaire is based on validated questions, where available, and has been piloted. Patients who agree to take part will leave the questionnaire with the receptionist.

##### Active

In addition to having their records flagged to allow opportunistic provision of the questionnaire, eligible patients in the Active arm will also be sent a postal questionnaire (with reply-paid envelope). If the questionnaire has not been received within four weeks after posting, then a first reminder will be sent. After a further four weeks, a second reminder will be sent. (It has been shown that the response rate to respiratory questionnaires is maximized with two reminders) [34].

Once the questionnaire has been returned to the investigators, the computer flag will be removed.

#### Stage 2: spirometry assessment

Any patient responding positively to the respiratory symptom questions (chronic cough or phlegm for three or more months of the year for two or more years, wheeze in the last 12 months or dyspnoea of MRC grade 2 or more) will be invited to attend for spirometry to ascertain whether they have COPD or not.

This will take place in a room at the patient’s GP practice or an alternative local venue. Post-bronchodilator spirometry will be undertaken according to ATS/ERS 2005 guidelines [35] and carried out by research assistants trained to high standards using a short modified programme modelled on the ARTP Spirometry course by the lung function unit at Queen Elizabeth Hospital Birmingham. The research assistants will undertake frequent refresher training and monitoring of quality throughout the trial. FEV_1_, FVC, FEV_6_ will be measured using the Easy One spirometer (ndd, Switzerland). Patients will be administered 400 micrograms salbutamol through a spacer, and asked to rest for 20 minutes prior to undergoing spirometry. Customised software (MMiller) will be used to ensure real-time quality of each manoeuvre and every trace over-read.

All spirometry results will be provided to their GP.

During the assessment visit, patients will also be asked to complete a short questionnaire to ascertain their out of pocket expenses for attendance (Additional file 1: Appendix 3), health status (EQ-5D) [36], job type (using CASCOT software) and the COPD Assessment Test. [37] Patients’ height will be measured to the nearest cm using a portable stadiometer (or estimated using arm-span if necessary), and weight (to the nearest 0.1 kg) will also be measured using GP scales by trained researchers.

### Allocation to trial arm

#### Cluster randomised trial comparing targeted case finding with routine care

Recruited practices will be allocated into intervention arms using block randomization. Ideally block size will be at least eight practices [38, 39], with an equal number of practices allocated to intervention and control groups by the end of each block.

A minimization algorithm will be used to allocate practices to intervention groups to ensure balance across the groups for the following covariates: deprivation, ethnicity, practice size, age and proportion of COPD patients on the Quality and Outcomes Framework (QOF) COPD register [40]. Any linked practices (those practices that have the same GPs across two sites, each with a different patient list) will be stratified so that the allocation to trial arm will be the same in order to avoid contamination. The algorithm generates an ordered list of the possible permutations of randomization within each block – at the top of the list are the permutations which minimize imbalance the best. Of those at the top of the list (e.g. the top 10%), one will be chosen at random to use.

#### Individually randomised RCT comparing active with opportunistic case finding

Within each practice randomised to the Targeted arm, each household will be further randomised to either active or opportunistic case finding. Thus in the event of multiple patients from the same household, they will therefore be allocated the same intervention group in order to avoid contamination. Allocation will be undertaken automatically by the research database as each practice list is uploaded.

### Outcomes

#### Primary outcomes

The primary outcomes are the percentage of the eligible population diagnosed as new COPD cases within the first year, and cost per additional case identified in each arm, comparing:

1. Targeted case finding and routine practice
2. Active and opportunistic case finding

#### Secondary outcomes

1. Feasibility (process measures such as uptake and resource needs).
2. Efficiency (number needed to target to identify one person likely to benefit).

### Diagnosing COPD

For the cluster part of the trial, patients in the routine arm may only be newly diagnosed with COPD during usual care and will be identified from GP records using the same method as that used to identify and exclude patients with a prior diagnosis of COPD in the initial search (Additional file 1: Appendix 1). The current NICE guidance is that patients with appropriate chronic respiratory symptoms must also meet spirometric criteria (FEV_1_/FVC < 0.7) [31]. Thus, in order to compare with routine care, our primary definition must be FEV_1_/FVC < 0.7 in the presence of respiratory symptoms. However this definition is controversial, and therefore, we propose also to use the “Lower Limit of Normal” (LLN) definition using the recently established GLI 2012 equations [41]. This requires that the post-bronchodilator FEV_1_/FVC ratio falls below the 5th percentile of the predicted values for patients’ age, height, sex and ethnicity. All patients must also report chronic respiratory symptoms. In sensitivity analyses we will explore other definitions such as use of different cut-offs for the LLN.

### Additional data collection

Data on the practices and patients will be collected at several stages during the trial:

#### Practice level information

Information on the characteristics of each of the participating practices will be collected, to include list size, number of eligible patients, number of existing COPD patients, deprivation score, rural/urban status, ethnicity profile, number of GPs, designated practice contacts and age profile of the practice.

#### Information on all eligible patients

At baseline, practices will provide an anonymised dataset on each of the eligible patients (in both routine practices and targeted arms) to allow comparison between the arms of the trial. To include: age, sex, ethnicity, smoking status, co-morbidities, health service use and reasons for exclusion.

At the end of the year, practices will also provide information on the number of consultations for each eligible patient, number of new cases of COPD identified outside or within the trial, whether patients have left the practice or have died, and among a sample, the number of flags remaining on the GP system to give an indication of the proportion of questionnaires distributed opportunistically.

### Statistical justification for sample size

As there are two primary outcomes, the significance level for multiple testing is adjusted so that in total (across both sample size calculations) there is a 5% significance level. The individually randomised component (outcome (b) below) comparing Active versus Opportunistic case finding requires far fewer patients for adequate power and is therefore given 0.25% significance level. The cluster component (a) is designated 4.75% significance level.

#### (a) Targeted case finding versus Routine care cluster randomized design

Based on the authors’ modelling work [32], the proportion of new COPD cases detected in the Targeted group (averaged across both Active and Opportunistic arms) was assumed to be 3.15%. Based research by Simpson et al. [42], the proportion of new COPD cases detected in the Routine care group was assumed to be 0.75%. At the 4.75% significance level, with 80% power, this led to an unadjusted sample size required of 545 per group, in order to detect a difference of 2.4% between Targeted and Routine care arms.

We expect ~40% of a practice population to be age 40-79 yrs with 57% of these being ever smokers without a previous diagnosis of COPD [32]. Assuming therefore a conservative 1000 eligible patients per practice of average list size 6000, and adjusting for clustering of patients within practices, assuming a conservative ICC of 0.05 [43, 44] the sample size required was 27,768 per arm, equivalent to 28 practices per arm.

#### (b) Targeted arm: Active versus opportunistic case finding

The following estimates are based on assumptions drawn from the authors’ previous modelling work [32]. We assume 50% allocated to the Active arm will respond; of the remaining patients 91% will visit their GP at least once in 12 months, 50% are offered the questionnaire and 90% of these will fill it out. In the Opportunistic arm we assume 50% are offered the questionnaire and of these 90% complete questionnaires (Figure 1). Of all responders to the questionnaire in both arms, we assume 48% are likely to report symptoms and be invited to spirometry, of which 70% will attend and 17% of these will have COPD. This leads to yield of 2.3% in the Opportunistic and 4.0% in the Active arms. At a 0.25% significance level, 3904 patients/arm are required to detect this difference with 90% power.

For the targeted case finding comparison, there will be 19,152 eligible in each arm, giving ample power for the primary comparison (based on above assumptions 448 new cases from Opportunistic arm and 771 from Active arm). This sample size provides additional power for secondary analyses and also allows more flexibility should the assumptions be more optimistic than anticipated.

### Statistics and data analyses

All statistical analyses will be undertaken in STATA version 13.

#### Primary analyses

There are two primary comparisons:

#### Targeted case finding vs Routine care (cluster design)

Baseline characteristics of patients and uptake rate at each stage will be described for each cluster, and compared across clusters to check for balance across groups. A multilevel logistic regression model will be used to model the probability of detecting a new case of COPD in the Targeted group compared with the Routine care group. Clustering of patients (by practice and household) will be accounted for using a multi-level structure. The data will also be modelled using a log-link and results from the logit-link and log-link models will be compared. OR and also RR and risk differences will be estimated [45, 46], as will the NNT (Number Needed to Target to identify one additional COPD case). The analysis will also adjust for variables used in the randomization (age, ethnicity, deprivation) which might affect probability of having COPD.

#### Within the targeted group, active case finding vs opportunistic case finding (individual patient design)

Baseline characteristics of patients will be compared for each arm (Active and Opportunistic) within each practice to check for balance. Clustering of patients within practices and households (if appropriate) will be accounted for using a multilevel logistic regression model. OR and also RR and risk differences will be estimated [45, 46], as will the NNT (Number Needed to Target to identify one additional COPD case). Between-practice heterogeneity in the effect of Active versus Opportunistic case finding will be examined, and if present, the range and variability of effects will be appropriately summarized.

#### Secondary analyses

In addition the following analyses are also of interest:

- Multilevel logistic regression will be used to compare each of the two components of the Targeted arm with Routine care
- Models will also be extended to estimate the effect of practice-level covariates on rates of COPD detection
- Interaction terms will also be included to assess whether patient-level factors such as age, sex, ethnicity, symptom profile and smoking history modify (interact with) treatment effects.
- The impact of clustering at the household level will be investigated in sensitivity analyses.

#### Economic analysis

The trial-based health economic analysis will consider two questions:

How cost-effective is a) an Active approach to case-finding and b) a simple Opportunistic approach compared with routine detection of COPD (routine care), in terms of cost per additional case detected?

A within-trial patient level analysis will be undertaken to determine the total and mean costs of case finding for each trial arm. An incremental cost-effectiveness analysis from a health care perspective will determine the cost per additional case detected, with a sensitivity analysis taking into account patient out of pocket costs in addition to health care costs. True cases of COPD detected by each method will be determined within the trial. The cost of case finding and spirometry will be established within the trial, and resource use data will be collected for each method and will include staff time, equipment, diagnostic testing and any consumables required. Data will be collected from a representative sample of practices to ensure generalisability. Unit costs (e.g. staff costs, equipment) will be collected from published sources. In addition a patient cost questionnaire will be administered to all who attend for spirometry to determine their out of pocket (travel and time) costs.

By using data from the trial which uses West Midlands’ practices only, there may be limits to the generalisability of the results of the trial. This will be explored within the economic evaluation using extensive sensitivity analysis. Key parameters will be varied to determine the impact of changes on results. Case finding in different patient sub-groups will also be considered. Non-parametric bootstrapping and probabilistic sensitivity analysis will be undertaken to explore uncertainty in the confidence to be placed on the results of the economic analysis and cost effectiveness acceptability curves presented.

#### Additional analyses

We also plan further related analyses not detailed here including:

1. A model-based cost-effectiveness analysis to consider longer-term effects of case-detection and subsequent treatment (cost per quality-adjusted life year gained)
2. A qualitative study exploring patients views of case-finding
3. A qualitative study exploring the views of healthcare professionals

### Patient advisory group

A patient advisory group (PAG) has been set up, chaired by Mr Michael Darby. This group is funded to meet at approximately quarterly intervals or according to need, and will advise on a range of aspects of the design, conduct, analysis and dissemination of the study. The PAG will discuss issues as requested by the CIs and the chair will report their comments back to the investigators.

### Data management

All data will be stored on a password-protected web-enabled customized database designed and hosted by the PC-CTRU. Paper-based information will be held in locked filing cabinets in the study office.

### Trial steering committee

We have established a Trial Steering Committee which will meet annually to advise the research team.

Chair: Prof Debbie Jarvis, Imperial

Statistician: Prof Simon Gates, Warwick

Health economist: Dr Jane Wolstenholme, Oxford

Patient representative: Mr Michael Darby

Investigators: Dr Rachel Jordan, Prof David Fitzmaurice, Prof Peymane Adab.

### Data monitoring committee

This is not a trial of a medicinal product and therefore does not require a data monitoring committee. However, the TSC will undertake some of this role.

### Interim analyses & stopping rules

There are no planned interim analyses or stopping rules as the study must run for a full calendar year in each general practice in order to allow the opportunistic intervention to occur.

### Regulatory issues

#### Ethics approval

The study has received ethical and research governance approval through the IRAS process. Ref: 11/WM/0403.

#### Patient consent

Patients receiving the initial screening respiratory questionnaire will receive a letter of invitation, and patient information leaflet (PIL) (Additional file 1: Appendix 4) and will provide consent by returning their questionnaire with the associated permissions and their contact details. Patients attending spirometry assessment will discuss the trial with the researcher and provide written consent to participate (Additional file 1: Appendix 5). Permission will also be sought to contact patients for future research studies.

#### Indemnity

The University of Birmingham holds the relevant insurance policy for this study.

#### Sponsor

The University of Birmingham will act as the main sponsor for this study.

#### Funding

National Institute for Health Research (NIHR) Programme Grants for Applied Research Programme (Grant Reference Number RP-PG-0109-10061).

**Start date:** July 2012

**Proposed reporting date:** July 2015

### Publication plan

The main trial paper reporting the effectiveness and trial-based cost-effectiveness analysis will be submitted initially.

## Discussion

This trial investigates the effectiveness and cost-effectiveness of two alternative modes of systematic case-finding for COPD compared with routine care. Previously undiagnosed patients aged 40 years and over with a positive smoking history will either receive a respiratory questionnaire when they routinely visit their surgery, or will also receive a questionnaire through the post. Those with indicative respiratory symptoms will be offered spirometry to diagnose COPD. There are many strengths of this study including: it is the first of this design based in a large sample of GP practices with a comparator group; it is set in a range of GP practices representative of urban UK; it has a pragmatic design which should reflect real life; the spirometry will be the best quality possible with highly trained staff and quality control; there will be a full cost-effectiveness analysis and also sensitivity analyses to reflect a range of scenarios; the effects will be explored across the range of GPs involved, allowing the cost-effectiveness in different types of GPs to be assessed. This trial should inform practice across the UK and elsewhere with similar healthcare systems, and help to direct current effort towards case-finding more efficiently.

However, a study of this size and complexity has many challenges. We will be coordinating a large number of practices, and as a screening trial, thousands of patients and patient data. Accurately identifying appropriate patients from GP databases with their complex coding is a known problem. It is not known how patients will respond to questionnaires, and whether (or how often) it is practical for GP staff to give out screening questionnaires during routine visits. These are some of the feasibility outcomes we will be measuring. Coordinating and arranging patient assessments with the dual issues of patient and GP capacity will also be a challenge, especially in the short time-frame. And finally, it will be important to discuss how this approach can be rolled out from a trial setting into routine practice.

## Authors’ information

Principal Investigators: Dr Rachel Jordan, Professor Peymane Adab, Professor David Fitzmaurice.

## Electronic supplementary material

Additional file 1: Appendix 1: READ codes used for identifying eligible patients. **Appendix 2.** Screening questionnaire. **Appendix 3.** Patient costs questionnaire. **Appendix 4.** Patient Information Sheet. **Appendix 5.** Patient consent form. (PDF 472 KB)

## Abbreviations

COPD: chronic obstructive pulmonary disease
NIHR: National Institute for Health Research.
