# Supplementary material for: TargetCOPD: a pragmatic randomised controlled trial of targeted case finding for COPD versus routine practice in primary care: protocol
Source: BMC Pulm Med. 2014 Oct 4;14:157. doi: 10.1186/1471-2466-14-157 (PMC4271517; doi:10.1186/1471-2466-14-157)
Supplement: Supplementary file 1 — Additional file 1: Appendix 1: READ codes used for identifying eligible patients. Appendix 2. Screening questionnaire. Appendix 3. Patient costs questionnaire. Appendix 4. Patient Information Sheet. Appendix 5. Patient consent form. (PDF 472 KB) [file 12890_2014_626_MOESM1_ESM.pdf]

Appendix 1 READ codes used for identifying eligible patients

**COPD inclusions (y)/ exclusions (N)**			
		**H3**	**Parent code for COPD**
Clinical system:EMIS Web and LV			
		**Child codes**	
	Y	H31	Chronic bronchitis
	Y	H32	Emphysema
	Y	H36	Mild chronic obstructive pulmonary disease
	Y	H37	Moderate chronic obstructive pulmonary disease
	Y	H38	Severe chronic obstructive pulmonary disease
	Y	H39	Very severe chronic obstructive pulmonary disease
	Y	H3A	End stage chronic obstructive airways disease
	Y	H3y	Other specific chronic obstructive airways disease
	Y	H3z	Chronic obstructive airways disease
	N	H30	Bronchitis unspecified
	N	H33	Asthma
	N	H34	Bronchiectasis
	N	H35	Extrinsic allergic alveolitis
Clinical system: System One			
		**Parent code**	
	Y	H3	COPD read codes in a cluster

**Smoking inclusions (y)/ exclusions (N)**			
		**137**	**Tobacco consumption Parent code**
Clinical system:EMIS Web and LV			
	**Included**	**Child codes**	
	Y	1372	Trivial smoker - < 1 cigarette per day
	Y	1373	Light smoker - 1-9 cigarettes per day
	Y	1374	Moderate smoker - 10-19 cigarettes per day
	Y	1375	Heavy smoker - 20-39 cigarettes per day
	Y	1376	Very heavy smoker - 40+ cigarettes per day
	Y	1377	Ex-trivial smoker - < 1 cigarette per day
	Y	1378	Ex-light smoker - 1-9 cigarettes per day
	Y	1379	Ex-moderate smoker - 10-19 cigarettes per day
	Y	137A	Ex-heavy smoker - 20-39 cigarettes per day
	Y	137B	Ex-very heavy smoker - 40+ cigarettes per day
	Y	137C	Keeps trying to stop smoking
	Y	137F	Ex-smoker - amount unknown
	Y	137G	Trying to stop smoking
	Y	137H	Pipe smoker
	Y	137J	Cigar smoker
	Y	137K	Stopped smoking
	Y	137M	Rolls own cigarettes
	Y	137N	Ex-pipe smoker
	Y	137O	Ex-cigar smoker
	Y	137P	Cigarette smoker
	Y	137Q	Smoking started
	Y	137R	Current smoker
	Y	137S	Ex-smoker
	Y	137T	Date ceased smoking
	Y	137V	Smoking reduced
	Y	137X	Cigarette consumption
	Y	137Y	Cigar consumption
	Y	137Z	Tobacco consumption NOS
	Y	137a	Pipe tobacco consumption
	Y	137b	Ready to stop smoking
	Y	137c	Thinking about stopping smoking
	Y	137d	Not interested in stopping smoking
	Y	137e	Smoking restarted
	Y	137f	Reason for restarting smoking
	Y	137g	Cigarette pack-years
	N	1371	Never smoked tobacco
	N	137L	Current non-smoker
	N	137I	Passive smoker
	N	137W	Chews tobacco
	N	137U	Not a passive smoker
	N	137D	Admitted tobacco consumption untrue?
	N	137E	Tobacco consumption unknown
Clinical system: System One			
	Y	Ub0oo	Tobacco smoking behaviour
	N	XE0oh	Never smoked tobacco
	N	137L	Current non-smoker

Appendix 2 Screening questionnaire

TargetCOPD POSTAL QUESTIONNAIRE TO PATIENTS

PATIENT ID________________

Thank you for taking the time to fill in this questionnaire. Your input is very valuable so please complete as many questions as you are able and return in the reply-paid envelope. Alternatively you may complete this form online at

https://www.pc-crtu.bham.ac.uk/Target

Please try to answer every question with the closest answer possible by ticking the appropriate box.

SECTION 1: YOUR LUNG HEALTH

1. (a) Do you usually have a cough (either during the day, or night, or first thing in the morning)?

   Yes ☐

   No ☐ **(If No, go to Q2)**

   (b) Do you usually cough like this on most days for 3 consecutive months or more during the year?

   Yes ☐ → If yes, for how many years have you had this cough? ....years

   No ☐

   (c) Does the weather affect your cough? Yes ☐ No ☐

2. (a) Do you ever cough up phlegm from your chest when you don't have a cold

   Yes ☐

   No ☐ **(If No, go to Q3)**

(b) Do you usually bring up phlegm from your chest (either during the day, or night, or first thing in the morning)? ☐Yes ☐No

(c) Do you bring up phlegm on most days for 3 consecutive months or more during the year?

Yes ☐ → If yes, for how many years have you had trouble with phlegm? .... years

No ☐

3. Have you had wheezing or whistling in the chest in the past 12 months?

   Yes ☐ → If yes, how frequently do you wheeze?

   Occasionally ☐ More often ☐

   No ☐

4. Are you troubled by shortness of breath when hurrying on level ground or walking up a slight hill?

   Yes ☐ No ☐

5. Do you get short of breath walking with other people of your own age on level ground or have to stop for breath after about 15 minutes when walking at your own pace?

   Yes ☐ No ☐

6. Do you have to stop for breath after walking about 100m or after a few minutes on level ground?

   Yes ☐ No ☐

7. Are you too breathless to leave the house, or breathless while dressing or undressing?

Yes ☐ No☐

8. Can you lie flat at night?

Yes ☐

No ☐ → If no, how many pillows do you need **in total**?.................................

9. Do you have or have you had any allergies?

Yes ☐

No ☐ **(If No, go to Q11)**

10. If yes, what type of allergies? (tick any that apply)

Hay fever ☐ Eczema ☐ Skin allergies ☐ Allergic rhinitis (nose/eye symptoms) ☐

Food allergies ☐ Other ☐ (please specify)……………………………………………

11. Do you usually have a blocked or running nose? Yes ☐ No ☐

12. Over the last year has your breathing kept you from doing as much as you used to? ☐Yes ☐No

SECTION 2: YOUR GENERAL HEALTH AND CIRCUMSTANCES

13. How would you describe your health in general?

Very good ☐ Good ☐ Fair ☐ Bad ☐ Very bad ☐

14. Has a doctor ever said you have (please tick any that apply):

Asthma	☐	High blood pressure	☐
COPD	☐	Diabetes	☐
Chronic bronchitis	☐	Stroke	☐
Emphysema	☐	Lung cancer	☐

Heart disease	☐	Tuberculosis	☐
Heart failure	☐	Depression	☐

Other medical condition (please specify)

………………………………………………

15. Have you ever had a paid job?

Yes ☐ Please state the occupation you have been employed in most of your life

……………………..

....

....

Please describe what you do/did in this job

…………………………………………………………………….............

......………………………………………………………………………

…………………………………………………………………………

…………………………………………………………………………

………………………………………………………….

No ☐

16. Have you ever worked in a job which exposed you to vapours, gas, dust or fumes?

Yes ☐

No ☐ **(If No, go to Q18)**

17. If yes, for how many years have you been exposed?

....

18. (a) Have you ever smoked as much as one cigarette a day (or one cigar a week or an ounce of tobacco a month) for as long as one year? ☐ Yes

No ☐ **(If No, go to Q19)**

(b) (b) How much do/did you smoke a day?

.............cigarettes/day ....cigars/week..........oz or ....g tobacco/week

(c) How old were you when you started smoking?....................

(d) Do you still smoke?

Yes ☐ **(If Yes, go to Q19)**

No ☐

(e) How old were you when you finally stopped smoking?......................

19. In most weeks, how many hours per week are you exposed to other people's tobacco smoke? ....

20. What is your current height without shoes? …… metres **or** ……feet..…inches

21. What is your current weight without shoes? …….kg **or**……stone…..pounds

22. Please indicate your date of birth: ……………………..

23. Sex: Male ☐ Female ☐

24. How would you class your ethnic group? (Please tick one)

White

English/Welsh/Scottish/Northern Irish/British ☐

Irish ☐

Gypsy/Irish Traveller ☐

Any other white background ☐

Mixed / multiple ethnic groups

White & Black Caribbean ☐

White & Black African ☐

White & Asian ☐

Other mixed ☐

Asian / Asian British

Indian ☐

Pakistani ☐

Bangladeshi ☐

Chinese ☐

Any other Asian background ☐

Black / African / Caribbean / Black British

African ☐

Caribbean ☐

Any other Black / African / Caribbean background ☐

Other ethnic group

Arab ☐

Other ☐

Prefer not to say ☐

SECTION 3: CONTACT INFORMATION

25. Title………………. First

name………...…………

Surname

…………………..…………………………..………......

26. Address………………………………………………………………

………………………………………………………………………………

………………………………………………………………………………

………………………………………………………………………………

……………..

27. Postcode………………………………............................………

………………

28. Telephone number

Home:………………………………............……………………

Mobile:……..........................………………………………………………

………………

29. Email

address……………………………………....……………………………

…

30. You may be invited for further assessment; to help us schedule these appropriately please indicate your preferred appointment times (tick any when you are available)

Monday	morning	☐	afternoon	☐	evening	☐
Tuesday	morning	☐	afternoon	☐	evening	☐
Wednesday	morning	☐	afternoon	☐	evening	☐
Thursday	morning	☐	afternoon	☐	evening	☐
Friday	morning	☐	afternoon	☐	evening	☐
Saturday	morning	☐	afternoon	☐	evening	☐

THANK YOU FOR TAKING THE TIME TO FILL OUT THIS QUESTIONNAIRE!

PLEASE RETURN AS INDICATED USING EITHER THE REPLY-PAID ENVELOPE OR

ONLINE AT

https://www.pc-crtu.bham.ac.uk/Target

Appendix 3 Patient costs questionnaire

Including patient costs in the TargetCOPD results

Please read the following information, which outlines why we are asking you to fill in this questionnaire.

The aim of the questionnaire:

Inviting people to attend spirometry (lung assessment) as part of a screening programme costs large sums of money. However, very little is known about the *hidden* costs of these assessments to the health service and to individuals taking part. An estimation of the costs would be incomplete if we did not consider the cost to the patients when attending for assessment. The information we get from this questionnaire will help us to find out this valuable information, and will be included in the TargetCOPD study results.

What you need to do:

We would appreciate it if you would take the time to fill in the questionnaire. It shouldn’t take longer than 5-10 minutes. Please answer **every** question in the best way you can.

STUDY ID: NAME:

Questionnaire for measuring costs to patients of attending a spirometry assessment clinic

Thinking about your clinic visit for spirometry:

1. Please enter today's date: ____/____/____

2. Where did your spirometry clinic take place? *(Please tick one box)*

Your GP surgery ☐

Other GP surgery ☐

Other ☐

If other, please specify ______________________________

3. What would have been your **main** activity if you had not attended the spirometry clinic?

Paid employment ☐

Looking after relatives ☐

Leisure activities ☐

Housework ☐

Studying at college ☐

Other ☐ *Please specify*________________

If you are in paid employment, please answer question 4, if not go to question 5

4. What arrangements did you make to take time off work? *(Please tick one box)*

Paid absence from work ☐

Unpaid absence from work ☐

Will make the time up ☐

Came to clinic outside work time ☐

Took holiday ☐

Other arrangements ☐

*Please specify*___________

5. How long did it take you to travel to the clinic?

________ hours ________minutes

6.

a) How did you travel to the clinic? Please tick the **main** (longest in terms of distance) form of transport.

Walking ☐

Private car ☐

Public transport - bus ☐

Public transport - train ☐

Taxi ☐

Other ☐ *Please specify* _______________

b) If you travelled by **private car**, were you given a lift by someone else?

Yes ☐ No ☐

c) If you travelled by **private car**, how much was paid in car park fees?

£_____p_____

d) If you travelled by **public transport (bus or train)**, what was the cost of the one-way fare? If you were given a return fare, simply halve it. Put zero if you did not travel by public transport at all or you did not pay a fare.

£_____p_____

e) If you travelled by **taxi** what was the cost of the (one-way) fare? Put zero if you did not travel by taxi at all or you did not pay a fare.

£_____p_____

7. Did anyone accompany you to the clinic **and wait** for you while you received your care?

Yes ☐ No ☐

If yes, did they take time off work? Yes ☐ No ☐

8. If you have other dependants,

Did you pay someone to look after them?

Yes ☐ No ☐ Not Applicable ☐

If yes, how much did it cost ? £_____ p______

or

Did someone take time off work to look after them ? Yes ☐ No ☐

9. How long did you spend waiting at the clinic **before** your appointment?

________ hours ________minutes

If you have any comments about your costs for attending the clinic or anything else about this study please write them below.

____

____

____

____

____

____

____

____

____

Thank you for your co-operation and help

Appendix 4 Patient Information Sheet

What is the study about?

TargetCOPD is a medical research trial designed to identify people who may have a lung disease called chronic obstructive pulmonary disease (COPD), also known as emphysema or chronic bronchitis. This is a common illness for which there is available treatment, although many people who have the illness don't know they do, and may not benefit from these treatments. We are writing to you because your GP has identified that you may benefit from an assessment. We would be grateful if you could complete and return the enclosed questionnaire. On receipt of your completed questionnaire we will decide whether you would benefit from having a routine lung test, known as spirometry, which is a simple breathing test and will be carried out by trained researchers.

Why is the study being done?

COPD is an increasingly important lung problem, leading to increasing breathlessness and sometimes other health problems. It affects about 1 in 20 people in the population. However, between half and three-quarters of these people are not aware that they are suffering from it, meaning that they may not get early treatment. The UK National Health Service has made identification of these undiagnosed patients a priority in its National Clinical Strategy for COPD, but we do not yet know how best to identify them. We think that this is an important study as it will test methods to identify patients at an earlier stage of their disease, allowing them to receive effective treatments, slow down the development of their disease and reduce their need for admission to hospital. Your help would be greatly appreciated.

What if I do not want to take part?

You are under no obligation to take part in this study; your medical care will not be affected in any way.

What will I have to do if I choose to take part?

The first stage is to complete the enclosed questionnaire about your respiratory health, your general health and personal circumstances. This should take about 10-15 minutes to complete. Please return it in the reply-paid envelope. You can also choose to do the questionnaire on-line: https://www.pc-crtu.bham.ac.uk/Target instead of completing the paper copy. Please log on and register using your ID number on the top of your letter.

On receipt of your completed questionnaire we will decide whether you would benefit from attending a further assessment at your GP practice or other convenient place. We will also ask your GP a few questions from your patient records.

What happens during the study assessment visit:

If you are invited for a lung test, the appointment should take approximately 45 minutes. During the visit we will collect information on, or measure:

1. Your lung health by simple blowing tests that measure the air you blow out (how much air and how fast), after breathing a common medicine (Salbutamol) given by an inhaler.
2. A short questionnaire about your respiratory health, general health, and family life style.
3. A short questionnaire about the costs to you of attending the assessment.
4. Other general measurements, such as your height, weight, and length of your arms.

We will also collect information from your GP notes on your medications and other medical details, which will be kept confidential. Finally, you may be invited to an interview to discuss your perceptions and experiences relating to the trial; this is completely voluntary.

What happens after the study?

We would like to follow up the health of everyone who agrees to take part directly through their medical and other health related records. Also, you may be invited to participate in other research studies and assessments with your permission. This will be entirely optional.

What are the possible disadvantages and risks of taking part?

Disadvantages of taking part are time and travel: you will be asked to fill out the initial questionnaire and possibly attend for one study assessment. If you choose to take part in the study then there is a small chance we may detect an abnormality in your lung test results that requires treatment. With your agreement we will pass these details onto your GP for further consideration. These are generally good things to be aware of as you may benefit from treatment.

It is possible that you may be slightly uncomfortable with some of the questions or measurements. If that is the case, you will have the option of skipping such questions or measures.

What are the possible benefits of taking part?

The information that we get from this study will help us to understand more about identifying COPD early. If you participate in the study, you will have better monitoring of your lung health and other aspects of your health. We hope you would find your

participation a rewarding experience and of course any costs you incur for travelling will be reimbursed.

What do I need to do now?

First of all, we ask if you would read this patient information letter thoroughly and decide whether you would like to participate or not. If you agree to take part, please return the questionnaire in the pre-paid envelope provided, as soon as possible. There is no need for you to add a stamp. Alternatively, you can reply on line at https://www.pc-crtu.bham.ac.uk/Target

What if I have more questions or do not understand something?

If you have more questions, or you do not understand something in this information letter, please contact our study team (details below). Alternatively, you can speak with your GP. If you would like independent advice on participating in research studies, you can contact the Patient Advice and Liaison Service (PALS) at Queen Elizabeth Hospital Birmingham (0121 371 3280, PALS@uhb.nhs.uk).

What happens now if I decide to take part?

Once we receive your reply, if you are chosen for further assessment we will contact you to schedule the visit.

What happens if I change my mind during the study?

You can withdraw from the study at any time without **giving a reason** and it will not affect your usual care from your GP in any way. If you decide to withdraw, **please** contact the study team (details below).

Do I need to agree to everything in the study?

No. If you feel uncomfortable with answering certain questions or do not want to have certain measures, you do not have to do so. You can still participate in the other parts of the study.

Will my taking part in the study be kept confidential?

All your personal identifying information (such as name and address) will be kept separately from other health and medical information we have for you. No one outside the study team can access your personal information. However, if you consent to take part in the research any of your medical records may be audited by people from regulatory authorities to check the study is being carried out correctly. Apart from these study activities, your name will not be disclosed outside the GP surgery. Your GP will be notified of your participation in the study. All data will be held on secure computers that block unauthorised access (e.g. by hackers) and are password protected.

Who is running the study?

This study is a joint study between the University of Birmingham, Queen Elizabeth Hospital and South Birmingham Primary Care Trust, with the cooperation of your GP. It is funded by the UK National Institute for Health Research through the Department of Health and has been approved by the Solihull Research Ethics Committee.

What happens to the results of the research study?

Once we have analysed the results we will publish them in a paper in a medical journal. We will also publish our results on our website

http://www.haps.bham.ac.uk/publichealth/research/bliss.shtml

You will not be identified in any publication.

Any queries or further information please contact:

Miss Ellen Sainsbury, BLISS Study administrator

HSRC Building, Public Health

University of Birmingham, Edgbaston,

Birmingham, B15 2TT

T: 0121 414 8348 bliss@contacts.bham.ac.uk

Website: http://www.haps.bham.ac.uk/publichealth/research/bliss.shtml

Thank you for taking the time to read this.

Appendix 5 Patient consent form

Study ID: **Practice ID**

Please initial boxes

I have read the attached information concerning my participation in this study and have had the opportunity to discuss and ask questions. All my questions have been answered in a satisfactory way and I give my consent voluntarily to participate in this study. ☐

I know that I can, at any time and without giving a reason, withdraw my participation in the study and that my future care and management will not be affected. ☐

I understand that information about me, where relevant to the study, will be stored by the BLISS research team at the University of Birmingham. ☐

I understand that personnel from the University of Birmingham research team may share my personal information with related NHS bodies and NHS regulatory authorities and may access my records for health related research purposes (even after my incapacity or death). ☐

I give my permission to authorise personnel from the research team to inform my General Practitioner of my participation in, and any relevant test results from this study. ☐

I give my permission to be contacted in the future for related research purposes including other studies in the BLISS programme. ☐

....	....	....
Name of Patient (CAPITALS)	Date	Signature
....	....	....
Name of Researcher (CAPITALS)	Date	Signature

If completed with a translator

I have provided an accurate translation of this information to the person mentioned above. They have stated that they understand the information and have had an opportunity to have their questions answered.

………………………………....... ……………... ....

Name of Translator (CAPITALS) Date Signature
